# Epidemiology and risk of juvenile idiopathic arthritis among children with allergic diseases: a nationwide population-based study

**DOI:** 10.1186/s12969-016-0074-8

**Published:** 2016-03-10

**Authors:** Chien-Heng Lin, Cheng-Li Lin, Te-Chun Shen, Chang-Ching Wei

**Affiliations:** Children’s Hospital of China Medical University Hospital, Taichung, Taiwan; Management Office for Health Data, China Medical University Hospital, Taichung, Taiwan; Department of Public Health, China Medical University, Taichung, Taiwan; Department of Internal Medicine, Division of Pulmonary and Critical Care Medicine, China Medical University Hospital, Taichung, Taiwan; School of Medicine, China Medical University, No2, Yu-Der Road, Taichung, 40402 Taiwan

**Keywords:** Allergic conjunctivitis, Allergic rhinitis, Asthma, Atopic dermatitis, Juvenile idiopathic arthritis

## Abstract

**Background:**

Epidemiological research reveals that the incidence of allergic diseases and that of autoimmune diseases have been increasing in parallel, raising an interest in a potential link between the two disorders. However, the relationship between Th2-mediated allergic disease and Th1-mediated juvenile idiopathic arthritis (JIA) remains unclear. This population-based case-control study was aimed at investigating the development of childhood-onset allergic diseases and the subsequent risks of JIA.

**Methods:**

We included 329 children with JIA diagnosed between 2000 and 2008, and 1316 age- and sex-matched controls. The odds ratios of developing JIA were calculated to determine an association with preexisting allergic diseases.

**Results:**

The incidence rate of JIA in Taiwan between 2000 and 2008 was 1.33 cases per 100,000 children/year according to the International League of Associations for Rheumatology (ILAR) criteria. The children with a single allergic disease had an increased risk of JIA, with adjusted odds ratios of developing JIA of 1.44 for allergic conjunctivitis (95 % confidence interval [CI], 1.07–1.95), 1.50 for allergic rhinitis (1.15–1.96), and 1.44 for asthma (1.00–2.10). The adjusted odds ratios increased with the number of concurrent allergic diseases from 1.50 (95 % CI, 1.12–2.01) for those with only one allergic disease to 1.72 (1.24–2.38) for those with at least two allergic diseases. The adjusted odds ratios of those with at least two allergic diseases increased to 1.84 (95 % CI, 1.19–2.86) for boys and 2.54 (1.42–4.54) for those older than 12 years. The children who made two or more medical visits for associated allergic diseases per year had an increased risk of JIA.

**Conclusion:**

Children with onset of allergic diseases were at increased risk of developing JIA. The increased risk was associated with the cumulative effect of concurrent allergic diseases and frequency of seeking medical care. Further study to investigate the role of Th2-mediated allergic diseases that contribute to the development of Th1-mediated JIA is warranted.

## Background

Juvenile idiopathic arthritis (JIA) is a chronic inflammatory disease in childhood that can lead to long-term morbidities such as uveitis, osteoporosis, depression, poor pain control, and even severe disability due to joint damage [1, 2]. JIA comprises a heterogeneous group of diseases, with chronic arthritis as a common feature [3, 4]. Although the etiology and pathogenesis of JIA are not fully understood, individual gene susceptibility and various environmental triggers (e.g. trauma, bacterial or viral infection, and hormone levels) may lead to an immune imbalance that subsequently results in articular and systemic manifestations [4, 5].

The autoimmune and inflammatory features of JIA appear complex. Children with JIA were found increased levels of activated CD4+ T-cells both in circulation and in the synovium [6–10]. In In oligo/polyarticular JIA, the activation of auto-reactive T-cells including Th1 and Th17 cells induced IFN-γ and IL-17 production, respectively, leading to the production of proinflammatory cytokines, chemokines and mediators such as IL-1, IL-6 and TNF-α [7–9]. On the other hand, the immunosuppressive function of regulatory T-cells (Tregs) with decreased anti-inflammatory cytokine IL-10 results in a loss of immune tolerance. Imbalance between auto-reactive Th1/Th17 and Tregs leads to the failure of T-cell tolerance to self-antigens, which contributes to synovial inflammation and subsequent joint damage [8–10].

Despite advances in the pathophysiology of JIA, the clear understanding of the initiating and perpetuating factors of JIA remains limited. Recent studies have revealed that the incidences of allergic and autoimmune diseases have been increasing in parallel, making them a serious health-care burden [11–13]. Interest in a complexity link between allergic and autoimmune diseases is increasing [13], as both involve chronic inflammatory processes with complex etiologies, influenced by some common genetic determinants or environmental factors. In contrast to Th2-mediated allergic diseases, JIA is a Th1-driven chronic inflammatory disease. However, human epidemiological studies evaluating the temporal interaction between Th1-mediated JIA and Th2-mediated allergic diseases are sparse. In addition, the epidemiological data of JIA in Asian countries are limited.

The Taiwan National Health Insurance Research database (NHIRD) provides unrestricted and universal access to health-care facilities for Taiwan’s population. In the present study, we used this claims dataset to investigate the incidence and clinical presentations of JIA, and define the influence of allergic diseases on the development of JIA so that we could provide better insights into the common immunological aberrancies of these conditions. We hypothesized that children with atopy have increased subsequent risk of JIA.

## Methods

### Data source

The NHIRD, maintained by the National Health Research Institutes, is population-based and derived from the claims data of the National Health Insurance program, a mandatory-enrollment, single-payment system created in 1995, now covering over 99 % of Taiwan’s population [14]. This file contained all medical claims and the information of insurant and provided a valuable resource, a unique opportunity and sufficient sample size to pursue the objectives addressed in this study. To ensure the accuracy of disease diagnosis, the National Health Insurance Bureau of Taiwan has randomly reviewed medical charts of 1/100 ambulatory and 1/20 inpatient claims. The high validity of the diagnostic data from the NHIRD has been reported previously [15]. Children file (age at <18 years) was derived from the NHI program and maintained by the National Health Research Institutes. Children file included 50 % children randomly selected from the Children Registry in 1996–2008 [15, 16]. This study was an analysis of de-identified secondary data; therefore, no informed consent was required. Although the identification information was scrambled, this study also received approval from the Institutional Review Board at China Medical University Hospital (CMUH104-REC2-115). Diagnostic code in the format of the International Classification of Disease, 9th Revision, Clinical Modification (ICD-9-CM).

### Subject selection

The study subjects were identified from two of sub datasets of the NHIRD: 1) Cases were identified from the Registry of Catastrophic Illnesses Patient Database (RCIPD), a dataset containing health claims data for treatment of catastrophic illness, which consists of thirty categories of diseases that require long-term care. The insured had major diseases such like JIA then they can apply for a catastrophic illness certificate. In Taiwan, the agreed-upon diagnosis and classification were the International League of Associations for Rheumatology (ILAR) revised classification, which refers to the illness as JIA. To reduce the financial hardship associated with catastrophic illness, the NHI program exempts beneficiaries from obligations for NHI-defined catastrophic illnesses. Cases subjects were patients aged less than 16 years-old, newly diagnosed with JIA (ICD-9-CM codes 714.0-714.9.0) between 2000 and 2008 as JIA group.

Thus, the accuracy of JIA cases identified from the RCIPD should be high; 2) Control subjects were identified from the Longitudinal Health Insurance Database 2000 (LHID 2000), a database containing the claims data of one million people randomly sampled from 2000 NHIRD enrollment file. There was no significant different in gender, age or health care costs between cohorts in LHID2000 and all insurance enrollees, as reported by the NHRI in Taiwan. For each JIA, 4 JIA-free controls frequency matched to the case on sex. We identified a total of 167 children with newly diagnosed JIA cases and 668 non-JIA controls.

We identified the subjects who were diagnosed with allergic conjunctivitis (ICD-9 codes: 372.05, 372.10, and 372.14), allergic rhinitis (ICD-9 code: 477), asthma (ICD-9 codes: 493), and atopic dermatitis ((ICD-9 code: 691.8) before the diagnosis of JIA. All diagnoses of the allergic disorders were defined as at least 3 respective ICD-9-CM codes in any diagnosis field of inpatient claim record or ambulatory claims. The validation of current study corresponding to previous studies, such as the International Study of Asthma and Allergies in Childhood (ISAAC), has been discussed in our previous paper [15]. The cumulative effect of disease severity was evaluated by the number of allergic comorbidities and frequency of seeking medical care. Both group with incomplete information were excluded.

### Statistical analysis

We used the chi-square and *t*-tests to analyze the demographic data between the JIA and non-JIA control groups, and multivariate logistic regression models to calculate the odds ratios (OR) and 95 % confidence intervals (CI) after adjusting for sex for the association between pre-existing allergic diseases and JIA. All data analyses were performed using SAS 9.1 (SAS Institute Inc., Carey, NC), and a *p* value less than 0.05 was considered to be statistically significant.

## Results

Table 1 shows the annual incidence rate of JIA among Taiwanese children aged <16 years. The mean annual incidence rate from 2000 to 2008 was 1.33 cases (range: 0.95–1.78 cases) per 100,000 children (Table 1). In total, 329 subjects with JIA were identified, including 161 boys (49 %) and 168 girls (51 %; Table 2). The prevalence of oligoarticular type JIA was higher in the boys (81 %) and children aged 6–12 years (54 %), whereas that of polyarticular type JIA was higher in the girls (56 %) and children aged 13–16 years (Table 2). The mean (SD) age at diagnosis of JIA was 11.3 (3.47) years. The prevalence of all atopic diseases was significantly higher in the JIA group than in the non-JIA group, including allergic conjunctivitis (22.2 vs. 16.6 %), allergic rhinitis (31.9 vs. 23.9 %), asthma (13.1 vs. 9.5 %), and atopic dermatitis (7.3 vs. 5.0 %), respectively (Table 3). The time interval (years) between the diagnosis of specific allergic disease and JIA was as follows: allergic conjunctivitis: mean = 3.86(SD = 2.30); allergic rhinitis: mean = 3.55(SD = 2.66); asthma: mean = 4.52(SD = 2.65); atopic dermatitis: mean = 3.09(SD = 2.47). In the whole study population, an increased subsequent risk of JIA was observed in the children with allergic conjunctivitis, allergic rhinitis, and asthma (Table 4). The adjusted odds ratios (aOR) were 1.44 for allergic conjunctivitis (95 % confidence interval [CI], 1.07–1.95), 1.50 for allergic rhinitis (1.15–1.96), and 1.44 for asthma (1.00–2.10). When the association was evaluated according to sex, the adjusted risk increased to 1.70 for allergic rhinitis (95 % CI, 1.15–2.50), 2.61 for atopic dermatitis (1.38–4.96) in girls (Table 4). When the association was evaluated according to age, the adjusted risk increased to 1.69 for allergic conjunctivitis (95 % CI, 1.03–2.76), 1.97 for asthma (1.19–3.25), and 1.95 for atopic dermatitis (1.05–3.62) in children younger than 12 years (Table 4). When the allergic patients were compared with those without any allergic diseases, the adjusted OR of JIA increased with the number of allergic comorbidities. The aOR increased with the number of allergic diseases, from 1.50 (95 % CI, 1.12–2.01) for those with only one allergic disease to 1.72 (1.24–2.38) for those with at least two allergic diseases (*p* value for trend < 0.0001; Table 4) in a multivariate logistic regression analysis. The adjusted ORs of JIA increased to 1.84 (95 % CI, 1.19–2.86) in boys and to 2.54 (1.42–4.54) in children older than 12 year (Table 4). We further examined the association between the annual frequency of medical visits due to allergic diseases and the risk of JIA, and found that the aORs increased for allergic rhinitis (2.27; 95 % CI, 1.42–3.63), asthma (2.05; 1.07–3.95), and atopic dermatitis (3.89; 1.19–12.7), with two or more medical visits per year (Table 5).

**Table 1:** Annual incidence of juvenile idiopathic arthritis (JIA) in Taiwan Children

Calendar year	Total population	JIA cases	IR
2000	2951131	36	1.22
2001	2909223	31	1.07
2002	2853181	27	0.95
2003	2794477	32	1.15
2004	2743320	34	1.24
2005	2697539	48	1.78
2006	2638402	36	1.36
2007	2569563	45	1.75
2008	2499236	40	1.60
Total	24656072	329	1.33

*IR* Incidence rate, per 100,000

**Table 2:** Demographics and subtype of juvenile idiopathic arthritis (JIA) in Taiwan Children between 2000 and 2008

	Total	Onset type of JIA		
		Oligoarticular	Polyarticular	Unspecified
	*n* =329	*n* =33	*n* =263	*n* =33
	n(%)	n(%)	n(%)	n(%)
Sex				
Male	161(49)	25(81)	117(44)	19(58)
Female	168(51)	8(19)	146(56)	14(42)
Age (year)				
<2	0(0)	0(0)	0(0)	0(0)
2–5	20(6)	2(4)	15(6)	3(9)
6–12	142(43)	20(54)	108(41)	14(42)
13–16	167(51)	11(42)	140(53)	16(49)

**Table 3:** Comparisons in socio-demographic factors and co-morbidities between cases with juvenile idiopathic arthritis (JIA) and controls

	Total *n* = 1645	non-JIA *n* = 1316	JIA *n* = 329	
	*n* %	*n* %	*n* %	*P*-value
Age (year), mean ± SD^a^	11.3 ± 3.47	11.3 ± 3.47	11.3 ± 3.47	0.95
Stratified age				0.99
<12	645 (39.2)	516 (39.2)	129 (39.2)	
≥12	1000 (60.8)	800 (60.8)	200 (60.8)	
Sex				0.99
Girls	840 (51.1)	672 (51.1)	168 (51.1)	
Boys	805 (48.9)	644 (48.9)	161 (48.9)	
Urbanization^b^				0.99
1 (highest)	495 (30.1)	396 (30.1)	99 (30.1)	
2	490 (29.8)	392 (29.8)	98 (29.8)	
3	270 (16.4)	216 (16.4)	54 (16.4)	
4 (lowest)	390 (23.7)	312 (23.7)	78 (23.7)	
Co-morbidity				
Allergic conjunctivitis				0.02
No	1354 (82.3)	1098 (83.4)	256 (77.8)	
Yes	291 (17.7)	218 (16.6)	73 (22.2)	
Allergic rhinitis				0.003
No	1226 (74.5)	1002 (76.1)	224 (68.1)	
Yes	419 (25.5)	314 (23.9)	105 (31.9)	
Asthma				0.06
No	1477 (89.8)	1191 (90.5)	286 (86.9)	
Yes	168 (10.2)	125 (9.50)	43 (13.1)	
Atopic dermatitis				0.10
No	1555 (94.5)	1250 (95.0)	305 (92.7)	
Yes	90 (5.47)	66 (5.02)	24 (7.29)	

Chi-square test

^a^ *t*-test

^b^The urbanization level was categorized by the population density of the residential area into 4 levels, with level 1 as the most urbanized and level 4 as the least urbanized

**Table 4:** Association between allergic diseases and risk for juvenile idiopathic arthritis

	All (*N* = 1645)	Girls (*N* = 840)	Boys (*N* = 805)	Age < 12 (*N* = 645)^a^	Age ≥ 12 (*N* = 1000)^a^
Allergic diseases	aOR (95 % CI)	aOR (95 % CI)	aOR (95 % CI)	aOR (95 % CI)	aOR (95 % CI)
AC	1.44 (1.07, 1.95)*	1.01 (0.63, 1.62)	1.92 (1.29, 2.87)**	1.69 (1.03, 2.76)*	1.33 (0.91, 1.96)
AR	1.50 (1.15, 1.96)**	1.70 (1.15, 2.50)**	1.36 (0.94, 1.97)	1.40 (0.93, 2.12)	1.59 (1.12, 2.26)**
Asthma	1.44 (1.00, 2.10)*	1.31 (0.74, 2.30)	1.57 (0.95, 2.58)	1.97 (1.19, 3.25)**	1.00 (0.56, 1.82)
AD	1.51 (0.93, 2.47)	2.61 (1.38, 4.96)**	0.74 (0.32, 1.71)	1.95 (1.05, 3.62)*	1.00 (0.43, 2.33)
Number of concurrent allergic disease					
0	1.00 (reference)	1.00 (reference)	1.00 (reference)	1.00 (Reference)	1.00 (Reference)
1	1.50 (1.12, 2.01)**	1.69 (1.13, 2.53)*	1.34 (0.88, 2.05)	1.12 (0.61, 2.07)	1.29 (0.71, 2.35)
2+	1.72 (1.24, 2.38)**	1.56 (0.95, 2.57)	1.84 (1.19, 2.86)**	1.42 (0.81, 2.50)	2.54 (1.42, 4.54)**
*P* for trend	<0.001	0.02	0.006	0.002	0.04

*Abbreviations*: *AC* allergic conjunctivitis, *AR* allergic rhinitis, *AD* atopic dermatitis, *OR* odds ratio, *CI* 95 % confidence interval

^a^The age is partitioned into two sub-segments (<12 vs. ≥12 years) based on mean age

*P < 0.05, **P < 0.01

**Table 5:** Association between frequency of annual medical visit due to allergic disease and risk for juvenile idiopathic arthritis^a^

Frequency	None	≤2	>2	*P* for trend
	aOR (95 % CI)	aOR (95 % CI)	aOR (95 % CI)	
Allergic diseases				
AC	1.00 (Reference)	1.12 (0.74, 1.72)	1.52 (0.81, 2.89)	0.20
AR	1.00 (Reference)	1.41 (0.93, 2.12)	2.27 (1.42, 3.63)*	<0.01
Asthma	1.00 (Reference)	1.45 (0.87, 2.39)	2.05 (1.07, 3.95)*	<0.01
AD	1.00 (Reference)	1.42 (0.74, 2.73)	3.89 (1.19, 12.7)*	0.02

*Abbreviations*: *AC* allergic conjunctivitis, *AR* allergic rhinitis, *AD* atopic dermatitis, *aOR* adjusted odds ratio, *CI* confidence interval

^a^Estimates from multiple logistic regression models adjusted for sex and age

*P < 0.001

## Discussion

Although the exact etiology of autoimmune diseases such as JIA remains unknown, it is believed to develop when a combination of genetic susceptibility and environmental encounters leads to the breakdown of tolerance [16–18]. Although methods for identifying JIA-susceptibility genes have advanced [19], only few studies have reported on the identification of environmental factors that trigger immunological aberrancies of JIA. Most of these studies described the associations between early-life infections, maternal smoking, and JIA [18–22]. Recently, three studies showed conflicting results on the association between atopic disorders and rheumatoid arthritis [23–26]. However, no reports have described the influence of childhood-onset allergic diseases on the risk of developing JIA. To the best of our knowledge, this is the first report on the increased risk of JIA in children with allergic diseases. The risk increased further with the accumulation of evidence on concurrent allergic diseases and the clinical burden of allergic diseases. Although our results only showed that children with allergic conjunctivitis, allergic rhinitis, or asthma were at significantly increased risk of developing JIA, the patients with other allergic disorders revealed increased frequency of JIA.

Epidemiological studies of JIA vary widely as regards to study methodology, classification criteria, and populations [27]. Comparison of epidemiological studies of JIA revealed that the annual incidence is 2.6 to 23 cases per 100,000 children per year and the estimated prevalence was 15.7–140 cases per 100,000 children [28–34]. The highest JIA incidence rates are found in some Scandinavian countries [30–32]. Studies from Asia revealed a low prevalence of JIA [35, 36]. The prevalence of JIA was estimated to be 3.8 cases per 100,000 children in Taiwan in 1995–1999 [35]. According to a smaller study from Japan, the annual incidence rate of JIA was estimated to be 0.83 case per 100,000 children in 1997 [36]. In this study, the mean annual incidence of JIA was 1.33 cases per 100,000 children in 2000–2008. All the JIA cases were obtained from the Registry for Catastrophic Illness Patient Database (RCIPD), a subpart of the NHIRD. In Taiwan, insured persons with major diseases can apply for a catastrophic illness certificate that grants exemption from copayment. The issuance of catastrophic illness certificates was validated by at least 2 specialists, based on careful examination of medical records, laboratory studies, and imaging studies. In Taiwan, the agreed-upon criteria for diagnosis and classification of JIA were based on the ILAR revised classification. Thus, the diagnostic accuracy for JIA should be high. However, the underestimated incidence of JIA based on data from the RCIPD should be considered because children with mild or oligoarticular disease may not apply or be qualified for a catastrophic illness certificate [37]. Although the most common subtype in Western European countries is represented by oligoarthritis, this is rare in countries such as Costa Rica, India, New Zealand, and South Africa, where polyarthritis predominates [27–30]. The present study revealed that the most common subtype of JIA in Taiwan was the polyarticular type. Previous studies from Asia showed that patients with oligoarticular JIA were older at disease onset and less likely to be female than their European counterparts, and that spondylarthropathy was more frequent in Asian patients [29]. Our study demonstrated a similar pattern that boys and children aged 6–12 years had higher prevalence rates of oligoarticular JIA.

JIA describes a clinically heterogeneous group of arthritis, which begins before 16 years of age. To date, the cause of the disease is still poorly understood but seems to be related to both genetic and environmental factors, which result in the heterogeneity of the illness. Our results support childhood-onset allergic diseases as risk factors of JIA. JIA is considered a complex genetic disease, and several genetic studies support that common aberrant immune responses may cause both JIA and allergic diseases. In Germany, Schubert et al. found an inverse association between several gene polymorphisms involved in Th1- and Th2-driven inflammatory pathways, such as IL-4, CTLA4, and TNF-α, and asthma and/or JIA [26]. In a Mexican population, Silvia et al. reported an association between the TNF alpha gene, a common factor in the pathogenesis of pediatric inflammatory and/or autoimmune diseases such as asthma, systemic lupus erythematosus, and JIA [32]. In Germany, Heinzmann et al. reported that an IL13 (Th2 cytokine) gene variant might prevent the development of JIA and make an individual susceptible to atopic diseases [33]. In addition to these genetic studies, common environmental triggers might be another explanation for the increased risk of JIA in atopic children. Kokkonen et al. found intestinal immune activation in most patients with JIA and observed similar features in children with delayed-type food hypersensitivity. This study implies that food-borne antigens might initiate pathological cascades in both disorders [34]. Recent evidence has suggested the role or dysfunction of Treg cells in the maintenance of immunological tolerance to environmental triggers and the prevention of autoimmune diseases and allergic diseases [31]. Treg cell dysfunction is undoubtedly connected to the two disorders; this supports our findings.

This study is the first to precisely analyze the future risk of JIA in children with common allergic diseases by using a large population database with minimal selection bias. However, it has some limitations. First, the diagnosis was based on the codes of the *International Classification of Diseases, Ninth Revision*. Hence, detailed clinical information such as anti-nuclear antibody titer, rheumatoid factor titer, IgE level, specific IgE, severity of arthritis and allergic symptoms, treatment response, susceptible genes, and environmental factors was lacking. Second, because JIA is a rare disease, the difference in related risk might be less significant or inconsistent depending on the limited sample size in the age- or sex-specific subpopulation analysis. Third, in this study, all the JIA cases were obtained from the RCIPD. Although the diagnostic accuracy for JIA should be high in the present study, the underestimated incidence of JIA from the RCIPD should be considered because children with mild or oligoarticular disease may not apply or be qualified for a catastrophic illness certificate [37]. In such cases, selection bias might exist, as the undiagnosed cases may be randomly selected as comparison controls. Fourth, not all patients with atopic disease would necessarily seek medical attention. These unrecognized and uncounted patients who were not included in the dataset will influence the results. Fifth, the subjects in this study were Chinese; thus, the study results cannot be generalized to other populations.

## Conclusions

In conclusion, children with onset of allergic diseases were at increased risk of developing JIA, and the increased risk was associated with the cumulative effect of the clinical burden of allergic diseases. Further study to investigate the role of Th2-mediated allergic diseases that contribute to the development of Th1-mediated JIA is warranted.

## Abbreviations

CI: confidence interval
ICD-9-CM: International Classification of Disease, 9th Revision, Clinical Modification
IFN-r: interferon-r
IL-1β: interleukin-1β
ILAR: International League of Associations for Rheumatology
JIA: juvenile idiopathic arthritis
NHIRD: National Health Insurance Research database
ORs: odds ratios
RCIPD: Registry of Catastrophic Illnesses Patient Database
TNF-α: tumor necrosis factor-α
